# Spasmodic Rhinorrhœa
*Introduction to a discussion at the Bristol Medico-Chirurgical Society on Wednesday, 12th May, 1939.


**Published:** 1939

**Authors:** E. Watson-Williams

**Affiliations:** Surgeon-in-Charge, Ear, Nose and Throat Department, Bristol Royal Infirmary; Clinical Lecturer in Diseases of the Ear, Nose and Throat, University of Bristol


### SPASMODIC RHINORRHŒA.*

BY

E. WATSON-WILLIAMS, M.C., CH.M.,

*Surgeon-in-Charge, Ear, Nose and Throat Department, Bristol Royal Infirmary; Clinical Lecturer in Diseases of the Ear, Nose and Throat, University of Bristol.*

---

THE condition to which I wish to direct attention is known by a variety of names—catarrh, coryza, rhinitis, rhinorrhœa or nasal hydrorrhœa, qualified as spasmodic, paroxysmal, intermittent, periodic, æstival, idiopathic, vasomotor, nervous, and, of course, allergic. As some of these names indicate, the characteristic symptom is an intermittent watery discharge from the nose. In addition there are often attacks of sneezing, irritation and obstruction of the nose, and lachrymation. These phenomena are, of course, seen as perfectly normal, physiological reflex reactions to any intranasal irritation; and they may be provoked by other stimuli such as bright light. An exaggerated response is probably more common than is usually supposed, being regarded, especially in winter or if the subjects are children, as "a slight cold." It is only when the attacks are sufficiently severe or prolonged that they are promoted to the dignity of disease.

The frequency of the attacks is very variable:

* Introduction to a discussion at the Bristol Medico-Chirurgical Society on Wednesday, 12th May, 1939.

it is easy to distinguish groups in which they are:—

(*a*) Quite irregular
(*b*) Perennial
(*c*) Periodic or seasonal.

Of the third class hay fever provides the best example. The essential similarity with the other two is evident from the frequent description as "hay fever when there isn't any hay." The following accounts illustrate many of the features:—

**Case 1.** (Irregular rhinorrhœa.) In 1922 I operated on the left antrum and ethmoid of a lady who attended occasionally since that time for inspection. Her general health improved very much after the operation, and remained good, and she was a particularly active and energetic woman. She never had any symptoms or evidence of disease in the right side of the nose until early this year, when she was 68: one afternoon as she sat at home she felt a sudden tickling in the nose and before she could reach for a handkerchief clear watery fluid began to pour from the right nostril. The attack ceased in half an hour, but a second occurred a week later, followed by others at decreasing intervals but of increasing severity, until they were occurring almost every day, and even when in bed at night, irrespective of what she was doing or whether she was at home or not. Her only warning was a tickling sensation, immediately after which the nose began to pour; in addition there were severe fits of sneezing, the cheek, nose and eyelids swelled up, with frontal and right-sided headache, lachryma-tion and even photophobia, and the right side of the nose became absolutely blocked. She might soak a dozen handkerchiefs in rapid succession and even then have dealt with but a fraction of the discharge, which was described as absolutely clear water, imparting neither colour nor the least stiffness or manifest change to the handkerchief. The severe attacks lasted three to four hours, and were not relieved by her doctor's ministra-tions: the nasal obstruction and headache persisted long after the other symptoms, her upper lip became badly excoriated, and she was reduced to a physical and nervous wreck for days after, when the attacks left her for so long. No cause at all could be found to explain the sudden change: after the attack had ceased I could discover no evidence of any disease in the right side of the nose, though the inferior turbinal was considerably more engorged than I had previously seen it : and except as compelled by the attacks she had not made any changes in her habits, diet or mode of life. Fortunately local treatment afforded complete and immediate relief.

**Case 2.** (Perennial rhinorrhœa.) I was consulted last September by a man of 32 complaining of "Hay fever every day for the last four months." He had never previously been affected and could think of no change in his work, habits or residence that might explain the condition : indeed, at first he had not worried much about it, regarding it as seasonal hay fever, until the persistence and increasing severity of the attacks brought him for advice. The attacks began with fits of severe sneezing lasting some minutes on end : they occurred always early in the day, and often actually woke him from sleep. After several sneezes the nose began to run clear water, and in a few minutes he might soak three or four handkerchiefs : the fluid did not mark these at all, but left them very slightly stiff. The later attacks lasted about an hour, leaving the nose obstructed most of the day. I found considerable swelling of the inferior turbinals but no other clinical or X-ray evidence of anything abnormal. Such a train of events always makes one suspect a new feather pillow, domestic pet or similar source of disturbance, but I have not been able to discover any such factor here.

### SYMPTOMS.

The symptoms shew great variations in relative intensity from case to case, but much less between the attacks which affect any one patient : they tend to become more severe in the later attacks.

*Irritation* is specially prominent in hay-fever, in which the patient may be conscious of a pricking sensation in eyes and nose for a day or two *before* the real attack begins. In all types it tends to increase during the early part of the attack. It may spread to the pharynx and larynx, causing great discomfort and a persistent, harsh, barking cough, usually without any visible changes in the larynx.

*Sneezing* is occasionally so severe as by itself to render the patient completely prostrate. There may be frequent paroxysms, or uncontrollable sneezing may continue for as much as an hour.

*The discharge* is always watery, never thick or purulent. It may literally pour from the nose: as much as six pints in a single attack has been recorded. Though very often apparently perfectly limpid, if examined in bulk it is faintly straw-coloured or opalescent, being in fact a very dilute mucus. It is faintly alkaline, and has the characteristic odour of mucus. Even when the mucin it contains is insufficient to stiffen a handkerchief, a chemical test (dilute acetic acid) shews it readily, and on heating the fluid we get a cloudiness due to coagulated albumen. It does not reduce Fehling's solution, a test which distinguishes it from the cerebrospinal fluid that drips from the nose in the rare and quite unrelated condition of cerebrospinal rhinorrhœa. It is usually sterile but contains many epithelial and eosinophile cells. Though it may appear quite innocuous, it is capable of causing very uncomfortable excoriation of nostrils and upper lip.

*Swelling* of the cheeks and eyelids may be extreme. I am inclined to regard the condition known as "Spasmodic Palpebral Œdema of Ethmoiditis" as merely a variant of the disorder under discussion, in which the œdema of the eyelids, often so severe as to close the eyes completely, is the prominent feature.

*Asthma* occurs in some cases, usually as the nasal symptoms subside; but it may replace the latter as the years follow on. It has been contended that it is caused by direct action of the exciting agent on the bronchi. The time factor suggests that the agent is not inhaled, but it might be conveyed by the circulation after absorption in the nose. The point is not important: there are many cases on record of asthma without any nasal symptoms, relieved by purely local treatment of the nose.

*General.*—Even with hay fever there is no real fever, or at most a very insignificant rise of temperature. But many patients experience a sense of physical and mental prostration after the attack out of all proportion to its severity. Bad temper and irritability are a phase of this.

*Examination.*—If one examines a patient during or just after an attack, the nose may present nothing abnormal beyond very pronounced swelling of the inferior turbinal. The latter is pale grey or violet in colour, sodden, flabby and usually in contact with the septum. It shrinks very poorly on the application of cocaine solution. When the attack has passed off the nose may appear absolutely normal, even in those who have been victims for years. But in some cases, though the appearance is normal, stroking the head of the inferior turbinal or the septum opposite the middle turbinal, which in normal persons would provoke merely sneezing and lachrymation, may suffice to start a typical attack. On the other hand, in those who have suffered long one frequently finds persistent pale hypertrophy of the inferior turbinal: perhaps less frequently polypi or other clinical or X-ray evidence of sinus disease, especially in the ethmoid. It used to be said that spasmodic rhinorrhœa might be for years the only symptom of latent sinusitis that eventually became manifest: I prefer the view that spasmodic rhinorrhœa may of itself initiate all the changes described. Septal spurs or irregularities may have some influence by making specially sensitive areas prominent, but they seem no more common in those who suffer from rhinorrhœa than in the population as a whole, and I believe are only occasionally significant.

### ÆTIOLOGY.

When the attacks are determined by personal idiosyncrasy to a substance of wide distribution, as in hay fever, they generally make their first appearance in adolescence. It seems probable that in many cases the early attacks occur in childhood but are relatively mild and apt to be overlooked: I have encountered a number of children under seven who suffered from typical seasonal hay fever. There is a well recognized tendency for the attacks to cease in later years. When they first appear at this period there is probably some additional factor to account for the development of susceptibility. In a few cases at any age a patient previously immune dates the commencement of his troubles to some illness such as influenza.

Among the factors that may induce an attack, we can recognize one or more of the following:—

(*a*) Local (nasal) supersensitiveness:
  1. General.
  2. Specific.

(*b*) An external exciting cause:
  1. *Specific: e.g.*, vegetable—pollen, orris-root; animal—emanation, food or extract.
  2. *Non-Specific:* dusts, vapours, cold, light, etc.

(*c*) Personal factors.
  1. Family tendency, illness.
  2. Habits—food, drink, toilet-preparations, medicines, occupation, etc.
  3. Endocrine.
  4. Nervous.

*Supersensitiveness.* General supersensitiveness of the nose, including the association with local abnormalities, has already been mentioned. Specific supersensitiveness is well illustrated in the case of hay fever, in which it is often the only factor of importance. In this country most havoc is caused by the grass pollens, which appear from the middle of May till July, the recognized "Hay Fever Season." Occasionally we encounter patients with an idiosyncrasy for trees such as the poplar, elm, etc., which flower in March, and in America the September-flowering docks are great offenders. The pollen of all these is wind-borne and often travels long distances, so that a susceptible person may be affected in the heart of a big city or even a mile or more out at sea. Certain conditions are necessary: on a cold day pollination does not take place, on a perfectly calm day the pollen is not distributed, heavy rain will wash the atmosphere clean, and of course a breeze directly off the sea is free from pollen. What are commonly called flowers, such as roses, are insect-fertilized, and their heavy pollen granules travel only short distances: the patients can therefore easily detect and avoid the cause of their trouble.

Flower pollens may appear in articles of diet. I have met with a patient, susceptible to rose-pollen, in whom attacks followed eating honey, probably collected from garden flowers, though he was not affected by really pure heather honey. I suspect too that the attacks ascribed to free indulgence in wine are due not to the alcohol but to the grape, though not to its pollen: but I admit chronic alcoholic excess as a cause of nasal turgescence. Another vegetable substance that is a frequent offender is orris-root, a component of many toilet powders: the more the patient sneezes the more often she must powder her nose and so start fresh paroxysms. Many articles of food have a definite seasonal appearance and attacks due to these may pass as hay fever. Other sources of trouble that may be difficult to detect are animal emanations, especially from feathers in pillows, bed-covers, etc. Some examples of idiosyncrasy will be given later.

Non-specific irritants are apt to be overlooked unless the time of the attacks points them out. Any irritant dust or vapour may start an attack. Quite recently I saw a man who had had to change his occupation because the irritation of powdered corundum used in grinding started severe spasms of sneezing and rhinorrhœa. He came to me because, after a period of freedom, his employers had set up a forge near his bench and the fumes brought a recurrence of his symptoms. In this case I think a very pronounced deviation of the septum is a factor of importance and I propose to see whether treatment of this will relieve him. Tobacco workers seem often affected, but only the "strippers" who work in an atmosphere made irritating by the dust of the raw untreated leaves: once the tobacco has been "cut" (*i.e.* steeped in alcohol) in the process of manufacture there is little trouble. It is a pity that the use of respirators where indicated in industry is not more general. Sometimes of course the stimulus is not nasal: typical attacks may follow exposure to bright sunlight, the sudden contact of the feet on a cold floor in the morning, or a draught of cold air—in the last case of course the patient insists that he has "caught a cold."

The possible influence of personal habits, occupation, diet, etc., has already been indicated. I should like to enquire how far the use of gas-fires is a factor of importance: in several cases I have thought it material.

Medicines may cause trouble. The effect of iodides on the nose is well known, but that of thyroid gland is not always appreciated. Cough mixtures containing terpene or similar substances affect many persons. The local application of drugs to the nose appears to be increasing. I have encountered many cases in which the nose became extremely sensitive following the prolonged use of Benzedrine inhalations, or irritating, useless disinfectant sprays and douches; and of late years the application of ephedrine in oil has become a positive family vice in many households.

*Endocrine System.*—The connection between the endocrine system and the nose has long been recognized. Patients taking large doses of thyroid gland may get quite severe rhinorrhœa; and the local application of adrenaline will produce a smart attack in many otherwise normal persons. Recently œstrogen has been used to increase nasal secretion in cases of atrophy. The association of sexual excitement with rhinorrhœa has been observed since the time of Trousseau, but appears to have attracted less attention than it merits. There are a few unfortunate men in whom every sexual excitement occasions a severe attack of rhinorrhœa. I have never met with such a case myself, but minor symptoms in males appear to be not uncommon. In women the premenstrual period is often accompanied by nasal irritability, at times amounting to troublesome rhinorrhœa. They are said, however, to be less often affected in this manner by sexual excitement. "Honeymoon catarrh" seems to be fairly frequent, but only rarely mars this felicitous period sufficiently to necessitate medical advice: not until I was going through my cases for this paper did

K

it strike me that three of the four cases I had seen, all quite severe, had occurred in the summer though the patients had never had hay fever.

*Nervous Causes.*—The influence of mental and nervous conditions is often very definite. It reaches perhaps its extreme form in those patients who, susceptible to the pollen of a particular flower, may suffer an attack on the sight of a similar artificial flower. Though this may be rare, it is common to find a patient who uses his disability to excite compassion or interest: the phrases "my hay-fever" or "my catarrh" frequently indicate an attitude of mind which requires treatment as much as the physical troubles.

Sometimes, of course, several factors combine. Thus in one patient of mine, in whom hay fever began with the menopause, not the endocrine change but thyroid medication for "slimming" was what determined the onset. In two tobacco-strippers symptoms were only really troublesome just before each period, though they never occurred except in connection with work.

### DIAGNOSIS.

In many cases the history of previous attacks, seasonal periodicity or association with some particular food or occupation enables the patient to make the diagnosis himself. It is the minor, atypical attacks or those occurring in winter that may be difficult. The watery, eosinophile character of the discharge and the absence of fever should prevent mistakes, and intradermal testing with extracts of suspected substances may confirm the diagnosis. Often, however, we shall fail to discover what actually does determine the attacks.

Perhaps* the coincidence of first onset with an

* *Lancet,* 1931. Vol. 2, p. 1237.

illness, change of residence or of occupation, supplies a valuable clue to the ætiology. But in other cases, although the periodicity or other circumstances point strongly to an occupational cause, the connection is quite obscure ; the attacks pursue the patient from one occupation or residence to another. Again, there may be no material change in occupation to account for the sudden appearance of attacks ; in these cases the patient is prone to ascribe them to shock, overwork, or nervous strain, recognizing clearly the "nervous" element in the causation. Before proceeding to dermal testing and desensitization, or even to operation, it is worth while to spend time in a careful investigation of the circumstances and of the mode of life of the patient. This may elicit some apparently trivial clue, on pursuing which the case becomes "Quite simple, my dear Watson !"

**Case 3.**—Miss A., aged 28, came to me in June, 1927, complaining of sneezing attacks, associated with intense watery nasal discharge, irritation, and watering of the eyes ; and during about a year nocturnal asthma, often sufficiently severe to wake her up. The attacks came on at any time of the day, and throughout the year, though they were worse in sunny weather. They had started four years' previously, and had at first been ascribed to hay-fever. She lived with her widowed mother, who kept a village shop. I inquired about animals ; they had had a cat and had kept poultry "for years" ; she did not think these could be concerned, for when she stayed with a sister who also had fowls she was quite free. Her own view was that the extra work entailed in looking after the shop and nursing her mother during a severe illness in the spring of 1923 had brought on the attacks. The nose appeared quite normal, but she had waited for a day when the attacks were better to come in to see me. Further questioning showed that from the time of her mother's illness she had taken over a large part of the domestic work, including feeding the fowls, collecting eggs, and cleaning the fowl-house. She agreed to get someone else to do this, and finding the attacks disappeared, gave up keeping poultry with complete relief.

**Case 4.**—Mr. C., aged 45, came to me in September, 1931, complaining of severe attacks of sneezing followed by intense nose-running, coming on every Sunday morning; by the evening he was almost prostrated, and felt very unwell all next day. The attacks had been going on for thirteen years, during which time he had been an ironmonger and a lorry driver in the North of England, later a gardener in Bristol, and now a cemetery attendant. He had now to work on Sundays occasionally, and the attacks came on as regularly then; the only time he was free was when he was in camp with the Territorials at the seaside—an inland camp did not help. Employers did not like a man who was always ill on Mondays, so he had become a teetotaller, except during the time when he was in camp. I wondered if beer was a cure, but he had had the same idea and given the suggestion sufficient trial to convince him of its futility. He nearly always spent his Saturday afternoons in his own garden; flowers would not be in season all the year round, but could it be bone manure? No, for the attacks came on even when he had not been in the garden. He had no dog, and had only had a cat for four years. The attacks started before church-time, so incense could not be blamed. The only difference from ordinary routine that applied to Sunday was that he got up two hours later, hardly a sufficient explanation. However, this supplied the clue; on Sunday he cooked himself a hot breakfast of bacon, on other days he had tea and cold food. I advised him to give up his bacon, a plan from which he was averse, for he was very fond of it, and it never gave him indigestion. He left me in much the same mood as Naaman the Syrian after his first visit to Elisha. Like Naaman, however, he resolved to give the simple prescription a trial. A month later I had a letter apologizing for not coming to see me again, "as it was quite unnecessary; I have not had another attack."

**Case 5.**—Miss B., aged 25, a wholesale chemist's assistant, came to me on 18th September, 1928, complaining of constant colds and violent sneezing attacks—"my nose simply pours." The attacks began rather suddenly nine months before, and she thought they must be due to intensive study for her final pharmaceutical examination, which she had passed in October, 1927; she had been working in the same dispensary for five years. The attacks were clearly connected with her work, as they never started on Sunday nor when she was on holiday. The obvious advice was to abandon her profession, but as usual this was not practical politics ; also, she had been dispensing for some years without trouble. Further questions elicited that the attacks usually came on about 4 p.m. and often lasted over the next day, seldom longer. They tended to be most frequent on Tuesdays and Fridays. The nose on examination appeared to be quite normal. The only clue to explain why the work she had done for so long without trouble should suddenly become noxious was the passing of the examination ! That is to say, she was now fully qualified, and employed in handling poisons, including "dangerous drugs," which had formerly not been part of her work. Tuesdays and Fridays were the days when "stocks" of these drugs were usually made up. Cocaine did not affect her. I advised that she should try leaving off the handling of opium and its derivatives, to which her employer agreed. For a month she was quite free from attacks. Further experiment showed that she could handle opium, extract of opium, tinctura opii, or liquor morphinae without suffering ; but whenever she had to put up powdered morphine sulphate or tartrate the attacks followed. Wearing a mask did a little to mitigate the distress. The only attacks she had after this could be directly traced to handling these drugs, usually when other dispensers were on holiday. When she left her employment to get married she remained entirely free from rhinorrhæa. There was a sequel to this case when four years later her employers asked her to work for them again because several of their staff were ill. This time it was necessary for her to do all the regular routine work and for the six weeks during which she was employed she suffered from her customary attacks whenever she handled morphine.

### TREATMENT.

In a limited number of cases it may be possible to detect and avoid the cause of the paroxysms. When this is almost universal, as in hay fever, avoiding it may entail a sea voyage or residence abroad, which few can afford. A more modern method is air-conditioning of sitting-room or office, a relatively inexpensive method. Now that the whole population is provided with gas masks it might be worth while to don one of these before the attack begins—one could hardly do so later. The great majority of patients want the shortest and simplest treatment that will enable them to live and to work as usual. As has been suggested, to obtain a good result it may be necessary to investigate and deal with a number of factors including diet, furniture and habits of life. And when, as in the first two cases I mentioned, it is impossible to discover any clue to the cause, prophylactic or specific treatment is impossible.

*General Measures.*—It is not my purpose to discuss general measures that should be adopted when a patient is first seen during an attack. It is wise to avoid both cocaine and adrenaline applications to the nose, the first on account of the danger of habit-formation, which is very definite in patients of this type, the second because the temporary relief is soon followed by an exacerbation of symptoms. A weak solution of cocaine alkaloid (not hydrochloride) in almond oil is not open to the same objections, as absorption is so slow that oxidation and excretion keep pace with it; alternatively a watery solution of tutocaine may be used: in either case a very small addition of ephedrine is permissible. But even a plain, bland oil spray has definite value. In hay fever bathing the eyes with a weak alkaline eye lotion is often comforting: it may be necessary to confine the patient to a room in which the window is closed so as to minimize exposure to pollen and severe photophobia may render darkness desirable. No medicines appear to have any specific effect, but bromides afford some measure of relief. Some patients find benefit from full doses of calcium by mouth.

*Desensitization.*—The first attempts at specific treatment were made with serum obtained from animals inoculated with various pollens. This method never received much success. Inoculation of the patient himself has a very definite value, especially in dealing with true seasonal hay fever. It is less useful where the patient reacts to numerous animal extracts, dusts and articles of food, etc. We must recognize that often we have to deal with patients who are "reactive" generally rather than particularly susceptible to one substance: they may give a vigorous skin reaction to almost every substance that is applied in the intra-dermal test; others of course give no reaction with any. The favourable patients are those who react to grass pollens, etc., but not to other substances. There is a certain amount of cross-sensitivity: that is, patients who are sensitive to one grass pollen may give dermal reactions with others. This is particularly noticeable in the Dock-Ragwort-Golden-Rod class that causes so much havoc in America.

For good results it is desirable to employ a large number of pollens and to make up for the patient a special vaccine from the information thus obtained. Stock preparations give good results in a much smaller proportion of cases. Treatment is best begun in the early part of the year well before attacks are to be expected. Beginning with very small doses, these are very cautiously increased at intervals of two or three days. Some thirty injections in all are required to reach an adequate dose without provoking reactions, and once this dose has been obtained it is continued, usually once a week, until the end of six months from the start and then once a month for a further eighteen months. Though this method is tedious, it is often successful even in cases of great severity. Desensitization can be applied when an attack is starting by carrying out inoculations every two hours, but requires special care in these circumstances. Anaphylactic shock is a possibility with any method of desensitization.

*Local Treatment.*—Obvious nasal abnormalities will naturally receive attention. Many of these patients suffer from disease of the sinuses, treatment of which is essential to a good result. Beyond this, local measures are designed to reduce the sensitivity of the nose, in the logical expectation of aborting the paroxysms. As a matter of interest, may I be allowed a glance at the history of some of these ?

The hardy Victorians sprayed or painted the interior of the nose with chemical caustics, employing strong solutions of mercury biniodide or perchloride, trichloracetic acid or silver nitrate. There is no question that these measures were usually successful, but morphine was always required for the intense pain, and unfortunately the sense of smell might be impaired or lost.

It was perhaps the desire for milder methods that inspired the procedures of Gottstein and of Zückerkandl towards the end of the last century. Gottstein's tamponade consists in the application of wool or gauze, variously impregnated, between the septum and the middle turbinal, intentionally avoiding the lower part of the nose. I remember how much time I spent when clinical assistant at the Golden Square Throat Hospital, just after the war, on this procedure. It is now called the "ethmoid pack." Zückerkandl practised massage, particularly of the inferior turbinal, with a rubber tube : rechristened

"diastolization," the method was revived some fifteen years ago.

Nasal ionization has had a still more chequered career. A standard textbook published in the early nineties mentions cataphoresis as a procedure which was losing support. About 1910 it came again into favour, but failed to survive the war. You will all remember the boom it enjoyed two years ago, largely, I believe, from the favourable notice of one of our large newspapers ; special clinics were even established for its administration. It is of course only a particular method of chemical cauterization, embellished with the mystery surrounding anything electric. Under local anæsthesia the olfactory cleft is carefully packed with tampons moistened with 1 per cent. zinc sulphate solution or jelly, surrounding zinc anodes : and a current of 5 milliampères is passed for five to fifteen minutes. However careful one is, it is very difficult to obtain in every case a coagulation uniform both in area and degree. Two or three sessions may be necessary and further courses may be required in subsequent years. Although there is no pain during application the immediate after-effects are extremely unpleasant : but a good result is sufficient compensation. Recent figures from a centre where large numbers are treated claim 18 per cent. cured and 45 per cent. improved : my own much smaller experience is considerably better than this.

The galvanic cautery has consistently maintained its reputation for fifty years. It can be applied very simply and accurately. The aim is to cauterize as lightly as possible the sensitive region, especially the septal crest, and as the local damage is very slight the reactions are minimal. The method has long enjoyed a reputation in the treatment of asthma

(unrelated to rhinorrhœa). Although it has often been said that the results are due to suggestion, they are at least as good as those of more elaborate or uncomfortable methods. The lady whose case I mentioned at the beginning of this paper was cauterized once, and although very sceptical was gratified by the complete cessation of her symptoms. It is one of the simplest and least unpleasant of the local measures and will probably continue in favour: the only drawback is that a certain degree of skill and experience are requisite for good results—too extensive or deep cauterization must be avoided.

*Summary of Treatment.*

1. During attack: local and general sedatives, calcium.

2. Prophylaxis:
   - (*a*) avoid cause if known;
   - (*b*) desensitization with vaccines, particularly in hay fever;
   - (*c*) treat nasal disorders, especially sinusitis (often unsuspected);
   - (*d*) local desensitization: actual cautery very lightly applied to special areas; in obstinate cases, despite difficulties and discomfort chemical coagulation (including ionization) often succeeds.

